# Epidemiology of overdose episodes from the period prior to hospitalization for drug poisoning until discharge in Japan: An exploratory descriptive study using a nationwide claims database

**DOI:** 10.1016/j.je.2016.08.010

**Published:** 2017-02-24

**Authors:** Yasuyuki Okumura, Nobuo Sakata, Kunihiko Takahashi, Daisuke Nishi, Hisateru Tachimori

**Affiliations:** a Research Department, Institute for Health Economics and Policy, Association for Health Economics Research and Social Insurance and Welfare, Tokyo, Japan; b Department of Biostatistics, Nagoya University Graduate School of Medicine, Nagoya, Japan; c Department of Mental Health Policy and Evaluation, National Institute of Mental Health, National Center of Neurology and Psychiatry, Kodaira, Japan

**Keywords:** Drug poisoning, Self-poisoning, Geographical variation, Benzodiazepines, Digitalis

## Abstract

**Background:**

Little is known about the nationwide epidemiology of the annual rate, causative substance, and clinical course of overdose-related admission. We aimed to describe the epidemiology of overdose episodes from the period prior to hospitalization for drug poisoning until discharge to home.

**Methods:**

We assessed all cases of admission due to overdose (21,663 episodes) in Japan from October 2012 through September 2013 using the National Database of Health Insurance Claims and Specific Health Checkups of Japan.

**Results:**

The annual rate of overdose admission was 17.0 per 100,000 population. Women exhibited two peaks in admission rates at 19–34 years (40.9 per 100,000) and ≥75 years (27.8 per 100,000). Men exhibited one peak in the admission rate at ≥75 years (23.7 per 100,000). Within 90 days prior to overdose, ≥60% and ≥9% of patients aged 19–49 years received a prescription for benzodiazepines and barbiturates, respectively. In addition, 59% of patients aged ≥75 years received a prescription for benzodiazepines prior to overdose, 47% had a history of congestive heart failure, and 24% had a diagnosis of poisoning by cardiovascular drugs. The proportion of patients with recent psychiatric treatments decreased with age (65.1% in those aged 35–49 years and 13.9% in those aged ≥75 years).

**Conclusions:**

The findings emphasize the need for overdose prevention programs that focus on psychiatric patients aged 19–49 years who are prescribed benzodiazepines or barbiturates and on non-psychiatric patients aged ≥75 years who are prescribed benzodiazepines or digitalis.

## Highlights

• Benzodiazepines were prescribed to ≥59% of overdose patients aged ≥19 years.
• Cardiovascular drugs were frequently ingested among overdose patients aged ≥75 years.
• The proportions of patients with recent psychiatric treatments decreased with age.
• Overdose prevention should be optimized according to the age-related differences.

## Introduction

Overdose episodes place a considerable burden on society and health care resources. Approximately 44,000 cases of death due to overdose occur annually in the United States, making it the leading cause of injury-related deaths.^1^ Non-fatal overdose is independently associated with subsequent overdose-related deaths.^2^, ^3^ Moreover, overdose was the major cause of emergency hospital admissions and the leading cause for admission to tertiary care centers in Japan.^4^ The direct medical costs of overdose have been estimated at 7.7 billion yen per year in Japan.^5^

The patterns and causes of overdose vary by country and region, which might reflect the availability of drugs and the prescribing practice in the population.^6^, ^7^, ^8^ In the United States, opioids, benzodiazepines, and antidepressants are the leading prescription drugs involved in overdose-related deaths.^9^ In the United Kingdom, heroin/morphine, opioids, and antidepressants are the leading psychoactive substances involved in drug-related deaths.^10^ In Japan, hypnotics-sedatives and antipsychotics are the leading prescription drugs involved in overdose-related deaths.^11^ Due to the low consumption rate of opioids in Japan,^12^ they are not among the leading causes of overdose-related deaths.^11^

The prevention of overdose requires careful and appropriate prescribing practices, which can be achieved through training of physicians and pharmacists.^13^ Understanding the country- and region-specific patterns of overdose is essential for the appropriate planning of prevention programs.^14^, ^15^ However, at present, there are serious limitations to our understanding of the epidemiology of overdose. To the best of our knowledge, only a few nationwide epidemiological studies have examined the rate, causative substance, and clinical course of overdose admissions.^16^, ^17^, ^18^, ^19^, ^20^, ^21^ Most of the studies focused primarily on the initial day of admission for overdose,^18^, ^20^, ^21^ or the period from the date of admission to discharge to home at the initial hospital.^16^, ^17^, ^19^ Hence, previous studies would have been unable to accurately quantify the clinical course of overdose, as they did not follow patients who were transferred from acute care hospitals to other medical institutions. Furthermore, even though overdose episodes may be primarily due to prescription drugs rather than over-the-counter or illicit drugs, information on the actual prescription history prior to overdose is scarce.^22^, ^23^ Therefore, in the present exploratory study, we aimed to describe the epidemiology of overdose episodes from the period prior to admission for overdose until discharge to home using a nationwide claims database.

## Methods

### Data source

We conducted an observational study using the National Database of Health Insurance Claims and Specific Health Checkups of Japan (NDB), developed by Japan's Ministry of Health, Labour and Welfare. Since its launch in April 2011, approximately 1.6 billion claims have been added annually to the NDB. As of October 2012, the NDB included all claims electronically issued from 99% of the hospitals (medical institution with ≥20 beds) in Japan^24^ — a country divided into 47 prefectures, with a population of 126 million.^25^ The NDB covers almost all patients who received medical care services under the universal health insurance system,^26^ except for the those availing medical services not covered under public health insurance. The claims data include clinical and procedural information, such as the patient identification number, institution identification number, prefecture code of the institution, sex, age, date of admission, date of discharge, procedural codes, diagnostic codes, and drug codes. After a review of our study protocol by the NDB expert council, we entered into a contract with the ministry to use a dataset extracted from the NDB for the purpose of the present study. We adhered to the guideline on the use of the NDB,^24^ based on which we were obligated to use the dataset only in a pre-specified secure room. We were subject to an on-site audit performed by an independent auditor to confirm adherence to the guideline. Our study was reviewed and approved by the institutional review board at the Institute for Health Economics and Policy. Because all patient records were de-identified prior to analysis, the review board waived the requirement for informed consent.

### Definition of overdose episode

We identified all hospital admissions for overdose from October 2012 through September 2013. Overdose was defined as an initial definitive diagnosis of drug poisoning (T360–T509 according to the International Classification of Diseases [ICD]-10 codes). These codes include any overdose caused by intentional or unintentional poisoning, as reported in previous studies.^22^, ^27^, ^28^ We excluded patients who were diagnosed with overdose after the date of admission.

An overdose episode was defined as the period from the date of admission for overdose to the date of discharge to home or the occurrence of in-hospital death. We included the first episode for each patient from October 2012 through September 2013 and followed all patients until the date of discharge to home, the date of in-hospital death, or the end of September 2014. Patients who were initially admitted to emergency care for the treatment of overdose and then transferred to a psychiatric hospital for the treatment of mental illness were considered as having a single overdose episode. To identify an overdose episode, we used the patient identification number recorded in the database. To increase traceability, we used the identification number generated from the insurance identification number, birth date, and sex (called “ID1”) rather than that generated from name, birth date, and sex (called “ID2”).

### Psychiatric treatments and chronic conditions prior to overdose

We identified whether patients received a prescription for psychotropic medications and treatment from a psychiatrist within 90 days prior to the overdose episode. We included 121 psychotropic medications classified as sedatives-hypnotics (subdivided into benzodiazepines, barbiturates, and others), antipsychotics, antidepressants, mood stabilizers, and anticonvulsants, according to a commonly used prescription handbook in Japan (see eTable 1).^29^ We also assessed the history of chronic conditions, including 17 conditions defined using the diagnostic codes of the Charlson Comorbidity Index.^30^ We identified whether a patients was diagnosed with a chronic condition prior to the overdose episode using ICD-10 diagnostic codes.

### Specific drugs involved in overdose

We identified the drugs that led to the admission for overdose using the ICD-10 diagnostic codes. These drugs were classified into 15 drug classes.

### Health service use and clinical course of overdose

We determined the wards to which patients with overdose were admitted on the initial day of hospitalization. We also identified whether patients were transferred to other medical institutions, such as psychiatric hospitals, after the initial admission to emergency care for the treatment of overdose. Finally, we determined the overall length of stay and in-hospital death during the period from the date of admission for overdose to the date of discharge to home or the occurrence of in-hospital death.

### Statistical analyses

First, the annual rate of overdose-related admissions per 100,000 population was calculated for the sex and age-sex subgroups. The number of overdose episodes was divided by the population estimates of the 2010 population census.^31^ We used age groups (0–11, 12–18, 19–34, 35–49, 50–64, 65–74, and ≥75 years) similar to those used in previous studies.^22^, ^32^ We calculated the 95% confidence intervals (CIs) for the rates by using the exact tail method for the Poisson distribution.^33^ Second, the age- and sex-standardized rate ratio (SRR) was calculated for each prefecture. The 47 prefectures were used as geographical units for analyses because these usually correspond to tertiary medical areas. The SRR enables the comparison of the rate for overdose-related admissions in a prefecture to that in Japan as a whole (reference). The empirical Bayes estimates for SRRs were obtained from a set of observed and expected number of overdose episodes using the Poisson-Gamma model along with moment estimators in the DCluster package in R.^34^ Moreover, we calculated the 95% equal-tail credible intervals (CrIs) for the SRRs using posterior distribution.^35^ Third, descriptive statistics by age groups were computed for variables prior to the overdose, drugs involved in the overdose, and the clinical course data. Cells with a count ≤9 are not reported according to the cell size suppression policy of the NDB.^24^ All data were analyzed using R version 3.2.2 (R Foundation for Statistical Computing, Vienna, Austria).

## Results

### Annual rate of overdose-related admissions

**Fig. 1:**
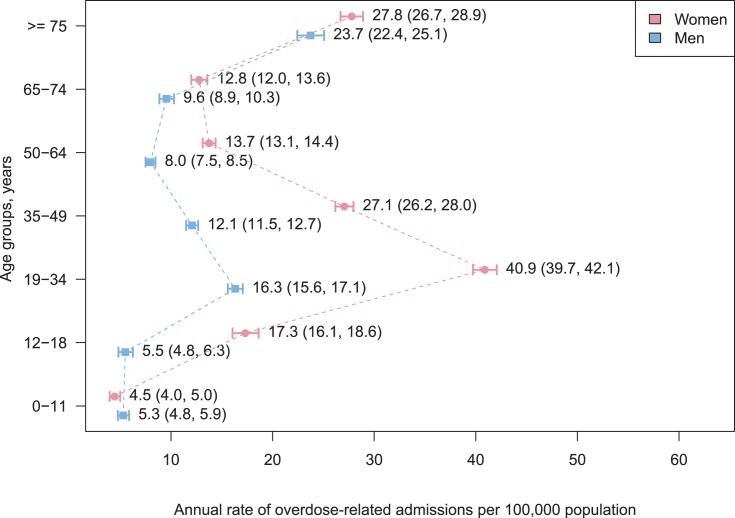
Sex- and age-specific annual rate of overdose-related admission.

**Fig. 2:**
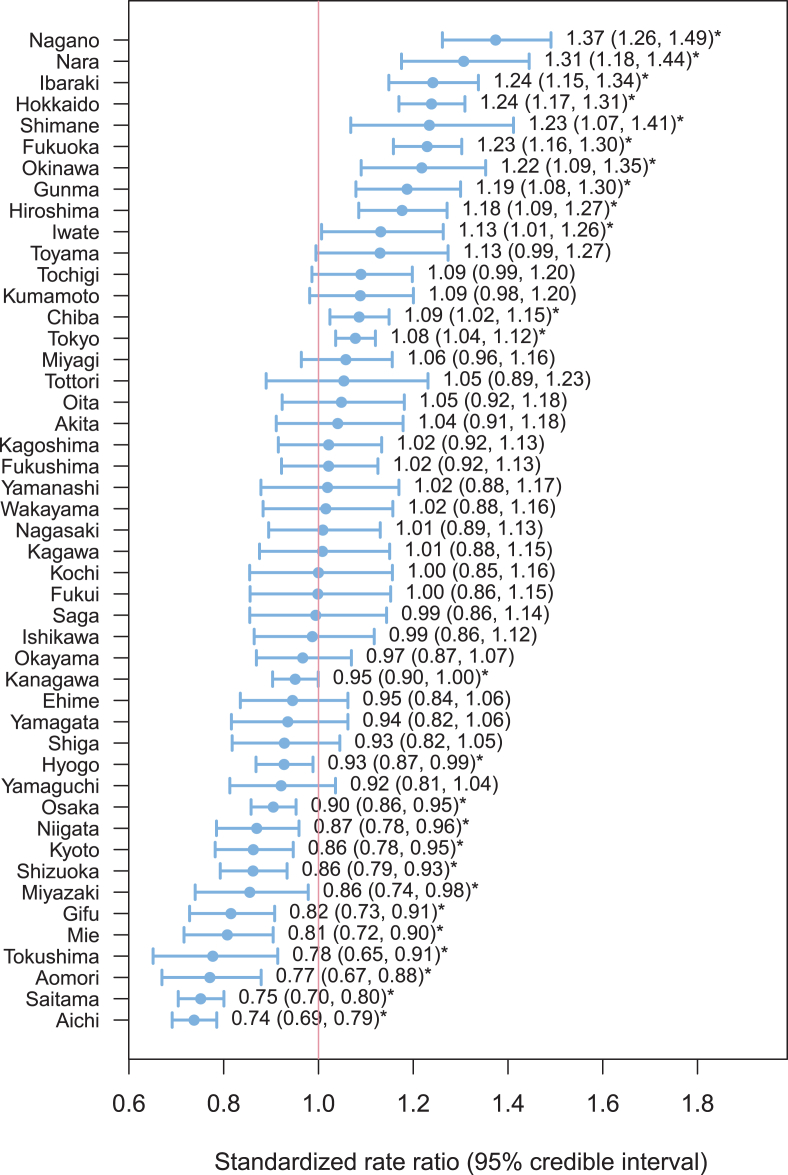
Age- and sex-standardized rate ratio with 95% credible intervals for each prefecture. * indicates statistical significance.

A total of 21,663 overdose-related admissions (14,543 women and 7120 men) in Japan were recorded in the database from October 2012 through September 2013. The annual rates of overdose-related admissions per 100,000 were 17.0 (95% CI, 16.8–17.3) for the overall sample, 22.3 (95% CI, 21.9–22.6) for women, and 11.5 (95% CI, 11.3–11.8) for men. The sex- and age-specific rate is illustrated in Fig. 1. Among patients aged 12–49 years, the annual rate of overdose-related admissions was 2.2- to 3.1-fold greater in women than in men. Women exhibited two peaks in admission rates at 19–34 years (40.9 per 100,000) and ≥75 years (27.8 per 100,000), whereas men exhibited one peak in the admission rate at ≥75 years (23.7 per 100,000). The geographical variation in the annual rate of overdose-related admissions is described in Fig. 2. The risk of overdose-related admissions was significantly higher in 12 prefectures as compared to that in the reference population. There was a 1.9-fold difference between the prefectures with the highest (Nagano) and lowest (Aichi) annual rates of overdose-related admissions (see eTable 2).

### Psychiatric treatments and chronic conditions prior to overdose

Within 90 days prior to the overdose episodes, 68.1% of the patients received a prescription for psychotropic medications, and 47.8% of the patients received treatment from a psychiatrist (Table 1). Sedatives-hypnotics were the most frequently used psychotropic medications (64.1%), followed by antipsychotics (34.6%), antidepressants (33.1%), and mood stabilizers (17.5%). Benzodiazepines were the most commonly used sedative-hypnotic medications (63.1%), followed by barbiturates (7.6%) and others (6.8%). With the exception of patients aged 0–18 years, the proportion of patients with a prescription for benzodiazepines was similar across age groups (range, 59.3%–73.8%), whereas the proportion of patients with a prescription for barbiturates peaked at 35–49 years (12.2%) and then decreased with age (1.2% at ≥75 years). In addition, the proportion of patients who received psychiatric treatment peaked at 35–49 years (65.1%) and then decreased with age (13.9% at ≥75 years).

**Table 1:** Prior history of chronic physical illness and psychiatric treatment.

		Age groups						
Prior history	Total, % (n = 21,663)	0–11 years (n = 647)	12–18 years (n = 949)	19–34 years (n = 6613)	35–49 years (n = 5182)	50–64 years (n = 2870)	65–74 years (n = 1708)	≥75 years (n = 3694)
Psychotropic prescriptions	68.1	8.0	45.0	67.9	76.6	73.5	73.1	66.5
Sedatives-hypnotics	64.1	4.2	37.5	63.6	74.2	70.6	69.1	61.0
Benzodiazepines	63.1	≤1.4	35.9	62.7	73.8	69.6	68.1	59.3
Barbiturates	7.6	≤1.4	2.6	9.0	12.2	9.4	4.2	1.2
Others	6.8	2.6	5.4	6.8	8.2	6.6	6.4	6.2
Antipsychotics	34.6	≤1.4	27.4	41.8	45.7	37.1	26.4	15.7
Antidepressants	33.1	≤1.4	16.8	41.4	45.7	34.4	24.3	13.9
Mood stabilizers	17.5	2.9	12.1	22.2	24.9	17.8	11.5	5.3
Anticonvulsants	9.1	4.5	5.4	11.2	11.9	9.2	6.9	4.2
Psychiatric consultation	47.8	≤1.4	40.4	61.0	65.1	51.5	32.7	13.9
Number of chronic conditions								
0	49.5	43.7	71.0	68.5	56.1	46.6	27.9	14.0
1	25.8	54.6	25.5	24.1	27.6	26.7	25.4	21.1
2	12.4	≤1.4	3.2	6.2	11.9	14.6	19.6	23.4
≥3	12.3	≤1.4	≤0.9	1.2	4.5	12.2	27.2	41.5
Chronic conditions								
Myocardial infarction	1.7	≤1.4	≤0.9	≤0.1	0.3	1.4	4.3	6.3
Congestive heart failure	11.4	≤1.4	≤0.9	0.8	2.6	7.8	19.0	46.5
Peripheral vascular disease	2.1	≤1.4	≤0.9	0.6	1.8	2.2	3.0	5.6
Cerebrovascular disease	11.9	≤1.4	≤0.9	0.9	2.8	10.6	27.3	43.2
Dementia	0.3	≤1.4	≤0.9	≤0.1	≤0.2	≤0.3	≤0.5	1.5
Chronic pulmonary disease	24.9	55.0	21.7	19.6	22.6	21.4	28.8	34.4
Connective tissue disease	2.5	≤1.4	≤0.9	0.8	2.0	4.4	4.6	5.2
Ulcer disease	22.6	≤1.4	8.1	14.7	22.2	25.4	33.6	37.4
Mild liver disease	4.2	≤1.4	≤0.9	1.4	4.4	6.7	7.0	7.6
Diabetes	2.1	≤1.4	≤0.9	0.3	1.5	3.1	5.7	4.9
Diabetes with end-organ damage	3.2	≤1.4	≤0.9	0.4	1.9	5.5	7.1	7.8
Hemiplegia	0.6	≤1.4	≤0.9	≤0.1	0.2	0.9	1.6	1.5
Renal disease	3.0	≤1.4	≤0.9	0.3	1.1	2.6	5.9	10.6
Any tumor	5.6	≤1.4	≤0.9	0.4	2.5	6.3	15.7	16.5
Moderate to severe liver disease	0.4	≤1.4	≤0.9	≤0.1	0.6	0.7	≤0.5	0.4
Metastatic solid tumor	0.9	≤1.4	≤0.9	≤0.1	0.3	1.5	3.2	2.4
AIDS	≤0.0	≤1.4	≤0.9	≤0.1	≤0.2	≤0.3	≤0.5	≤0.2

Cells with counts ≤9 cannot be reported according to the cell size suppression policy of the database. Following proportions were calculated for all cells using the actual numbers, proportions for the cells with counts ≤9 are displayed with the numerators equal to 9 along with the ≤signs.

Prior to the overdose episodes, 25.8% of patients had one chronic condition, 12.4% of patients had two chronic conditions, and 12.3% of patients had three or more chronic conditions. Chronic pulmonary disease was the most prevalent (24.9%), followed by ulcer disease (22.6%), cerebrovascular disease (11.9%), and congestive heart failure (11.4%). The proportion of having chronic conditions increased with age. Among patients aged ≥75 years, congestive heart failure was the most prevalent condition (46.5%), followed by cerebrovascular disease (43.2%), ulcer disease (37.4%), and chronic pulmonary disease (34.4%).

### Specific drugs involved in the overdose

Among the causative drugs defined using the ICD-10 diagnostic codes, unspecified drugs were responsible for the highest proportion of overdose admissions (T50, 64.0%), followed by anticonvulsants, sedatives-hypnotics, and antiparkinson drugs (T42, 21.9%); other psychotropic drugs (T43, 6.0%); and cardiovascular drugs (T46, 4.9%) (Table 2). The proportion of overdoses due to sedative-hypnotic drug use (T42) was similar across all age groups and ranged from 14.8% to 27.1%. Moreover, the proportion of overdoses due to cardiovascular drug use (T46) markedly increased with age and ranged from 0.2% in those aged 35–49 years to 24.3% in those aged ≥75 years.

**Table 2:** Ingested drugs that led to overdose-related admissions in the population.

		Age groups						
Ingested drugs (International classification of diseases-10 codes)	Total, % (n = 21,663)	0–11 years (n = 647)	12–18 years (n = 949)	19–34 years (n = 6613)	35–49 years (n = 5182)	50–64 years (n = 2870)	65–74 years (n = 1708)	≥75 years (n = 3694)
Systemic antibiotics (T36)	0.1	≤1.4	≤0.9	≤0.1	≤0.2	≤0.3	≤0.5	≤0.2
Other systemic anti-infectives and antiparasitics (T37)	≤0.0	≤1.4	≤0.9	≤0.1	≤0.2	≤0.3	≤0.5	≤0.2
Hormones and their synthetic substitutes and antagonists, not elsewhere classified (T38)	0.2	≤1.4	≤0.9	≤0.1	≤0.2	≤0.3	≤0.5	0.3
Nonopioid analgesics, antipyretics, and antirheumatics (T39)	3.5	7.4	12.6	5.4	2.5	1.7	1.5	0.9
Narcotics and psychodysleptics (hallucinogens) (T40)	0.4	≤1.4	≤0.9	0.6	0.3	≤0.3	≤0.5	≤0.2
Anesthetics and therapeutic gases (T41)	0.1	≤1.4	≤0.9	≤0.1	≤0.2	≤0.3	≤0.5	≤0.2
Anticonvulsants, sedatives-hypnotics, and antiparkinson drugs (T42)	21.9	18.5	14.8	19.9	21.9	26.2	27.1	22.0
Psychotropic drugs, not elsewhere classified (T43)	6.0	2.8	4.7	7.5	7.5	5.9	4.4	2.8
Drugs primarily affecting the autonomic nervous system (T44)	0.4	≤1.4	≤0.9	≤0.1	≤0.2	≤0.3	≤0.5	1.1
Primarily systemic and hematological agents, not elsewhere classified (T45)	1.8	3.4	1.2	0.4	0.5	2.1	5.4	4.2
Agents primarily affecting the cardiovascular system (T46)	4.9	5.3	≤0.9	≤0.1	0.2	0.8	4.7	24.3
Agents primarily affecting the gastrointestinal system (T47)	≤0.0	≤1.4	≤0.9	≤0.1	≤0.2	≤0.3	≤0.5	≤0.2
Agents primarily acting on smooth and skeletal muscles and the respiratory system (T48)	0.7	2.9	1.1	0.4	≤0.2	≤0.3	0.7	2.1
Topical agents primarily affecting the skin and mucous membrane and by ophthalmological, otorhinolaryngological and dental drugs (T49)	0.1	≤1.4	≤0.9	≤0.1	≤0.2	≤0.3	≤0.5	≤0.2
Diuretics and other and unspecified drugs, medicaments and biological substances (T50)	64.0	56.4	68.8	70.3	71.6	65.8	57.3	43.8

Cells with counts ≤9 cannot be reported according to the cell size suppression policy of the database. Following proportions were calculated for all cells using the actual numbers, proportions for the cells with counts ≤9 are displayed with the numerators equal to 9 along with the ≤signs.

### Health service use and clinical course of overdose

On the initial day of hospitalization, 36.5% of patients were admitted to the intensive care unit (Table 3). During the admission, 1962 patients (9.1%) underwent inter-hospital transfer. Of the transferred patients, 1242 patients (63.3%) were admitted to psychiatric wards in other hospitals. Over half of the patients (57.4%) were discharged to home within 3 days. The in-hospital death rate was 2% during the hospitalization for overdose episodes. The proportion of patients with a length of stay ≥31 days increased with age and ranged from 4.5% in those aged 12–18 years to 21.2% in those aged ≥75 years. Moreover, the in-hospital death rate increased with age and ranged from 0.5% in those aged 19–34 years to 6.5% in those aged ≥75 years.

**Table 3:** Information on admission, transportation, and clinical course of the patients who experience an overdose.

		Age groups						
Characteristics	Total, % (n = 21,663)	0–11 years (n = 647)	12–18 years (n = 949)	19–34 years (n = 6613)	35–49 years (n = 5182)	50–64 years (n = 2870)	65–74 years (n = 1708)	≥75 years (n = 3694)
Admission on the initial day								
General wards	54.3	97.4	55.5	45.3	45.4	52.1	61.1	73.4
Intensive care unit	36.5	2.2	36.1	44.7	44.2	36.2	29.0	20.7
Psychiatric wards	6.9	≤1.4	5.9	8.2	8.3	9.1	6.1	2.6
Others	2.4	≤1.4	2.4	1.8	2.1	2.6	3.7	3.4
Hospital transfer during the episode								
None	90.9	99.8	94.7	93.1	90.6	86.7	88.6	89.4
Psychiatric wards	5.7	≤1.4	3.8	5.0	7.4	9.6	6.3	2.9
General/other wards	3.3	≤1.4	1.5	1.9	2.0	3.7	5.1	7.7
Length of stay during the episode, days								
1	10.4	3.7	10.9	14.4	13.0	9.3	6.8	3.3
2–3	47.0	84.4	59.7	56.9	51.1	41.7	34.8	23.5
4–7	16.1	9.1	19.1	14.9	15.2	15.9	18.4	19.2
8–30	14.6	2.3	5.8	7.9	10.2	15.5	22.1	32.8
≥31	11.9	≤1.4	4.5	5.9	10.6	17.7	17.9	21.2
Death during the episode	2.0	≤1.4	≤0.9	0.5	1.0	1.7	3.0	6.5

Cells with counts ≤9 cannot be reported according to the cell size suppression policy of the database. Following proportions were calculated for all cells using the actual numbers, proportions for the cells with counts ≤9 are displayed with the numerators equal to 9 along with the ≤signs.

## Discussion

To the best of our knowledge, this is the first study to investigate the nationwide epidemiology of overdose episodes for the period from prior to admission for overdose until discharge to home. In Japan, 21,663 patients were hospitalized for overdose within a 1-year period, resulting in an annual rate of 17.0 per 100,000 population. The present results extend those of previous studies by demonstrating the actual number of overdose-related hospital admissions in a year in Japan.^4^, ^36^ In a report from the Patients Survey, the estimated number of discharged patients for overdose was 2100 in September 2014 (equivalent to 25,200 annual discharged patients) using a stratified random sampling procedure of medical institutions in Japan.^36^ Using a database from 855 acute-care hospitals in Japan, 6748 patients were discharged after admission for overdose from July through December, 2008 (equivalent to 13,496 discharged patients annually).^4^ However, the inconsistent definitions in various studies did not allow for a direct comparison of the findings among countries. For instance, the number in the United States (232 visits/100,000 population) is 13.6-fold higher than that in Japan; however, this value in the United States reflects the number of emergency department visits for overdose rather than that of hospital admissions.^19^ Moreover, compared to that in Japan, the number is 6.8-fold higher in New Zealand (115 admissions/100,000 population),^18^ 5.1-fold higher in Taiwan (86 admissions 100,000 population),^20^ and 9.8–12.4-fold lower in Korea (1.4–1.7 admissions/100,000 population).^16^ Nonetheless, these values reflect the number of admissions for poisoning by drugs and other chemicals (i.e., alcohol, carbon monoxide, and pesticides) rather than that for poisoning by drugs alone.^16^, ^18^, ^20^

In the present study, there was a 1.9-fold difference between the prefectures with the highest and lowest annual rates of overdose-related admissions. Information on the regional variation in the annual rate for overdose admissions is scarce in the literature.^6^, ^37^ One possible explanation for this variation is that the regional prescribing pattern of a specific drug leads to negative consequences of its use, such as overdose-related morbidity and mortality, in that population.^6^, ^15^ Since 2013, each regional government in Japan has a mandatory role in monitoring health care indicators, such as the number of patients with five diseases (cancer, stroke, acute myocardial infarction, diabetes, and mental disorders), five services (emergency medical care, medical care in disasters, medical care in remote areas, perinatal medical care, and pediatric medical care), and in-home medical care.^38^ Regional governments might provide public health approaches for overdose prevention, such as regulating and monitoring of prescription drugs, educating practitioners and the health care community regarding prescription drug abuse, informing patients and their families about the risk of prescription drugs, and making it easier to dispose of unused tablets.^13^, ^39^ Our results provides foundation for each regional government to monitor the regional overdose patterns and to ensure appropriate planning of region-specific overdose prevention programs.^14^, ^15^

We observed that ≥61% of patients aged 19–49 years had already received treatment from a psychiatrist prior to overdose admission. Although our data cannot be used to identify the intention of overdose, these patients were more likely to have intentional overdose rather than unintentional overdose. This is because the proportion of patients who receive psychiatric treatment for intentional overdose is as high as 80%,^40^ and the annual admission rate for intentional overdose is much higher in this age group than that for unintentional overdose.^18^, ^19^ In addition, we observed that ≥60% and ≥9% of patients aged 19–49 years received a prescription for benzodiazepines and barbiturates, respectively, prior to overdose admissions. The use of benzodiazepines and barbiturates increases the risk of overdose.^22^, ^23^ Furthermore, barbiturate overdose increases the risk of a severely adverse clinical course, including aspiration pneumonia and death.^11^, ^41^ Therefore, these findings emphasize the need for psychiatrists to carefully evaluate the risk of overdose for those aged 19–49 years who have a prescription for benzodiazepines and to consider treatment alternatives for those with a prescription of barbiturates.

We also observed that only 14% of patients aged ≥75 years already received treatment from a psychiatrist prior to overdose admission. These patients were more likely to experience an unintentional overdose rather than an intentional overdose, because the proportion of patients receiving psychiatric treatment rate for intentional overdose is as high as 80%^40^ and the annual admission rate for unintentional overdose is much higher in this age group than that for intentional overdose.^18^, ^19^ Benzodiazepines and digitalis may be the most commonly ingested drugs among elderly patients. These hypotheses are supported by the fact that 59% of elderly patients already received a prescription for benzodiazepines prior to admission for overdose, 47% had a history of congestive heart failure, and 24% had a diagnosis of poisoning by cardiovascular drugs. In addition, we observed peak annual rates of overdose admissions at ≥75 years old in both women and men. Elderly patients are more likely to have several risk factors for unintentional overdose than younger patients, including medication non-adherence caused by complex treatment regimens,^42^ impaired hepatic and renal drug clearance,^43^ and use of multiple prescribers to obtain prescriptions for the same medications.^44^ These findings further emphasize the need for non-psychiatrist physicians to improve the monitoring of serum levels and medication adherence in elderly patients with a prescription for digitalis.^45^ In addition, there is a need to promote the judicious prescription of benzodiazepines and the consideration of treatment alternatives, as the prevalence of benzodiazepine use in elderly patients is much higher in overdose patients (59%) than that reported in general practice (approximately 25%).^32^ To prevent overdose episodes, elderly patients with a prescription for benzodiazepines may certainly benefit from regular monitoring of medication adherence and side effects and adjusting of drug dosage.

Our study had several limitations. First, our results should be interpreted in terms of inpatient service admission due to overdose, so they do not reflect episodes of less severe overdose (i.e., outpatient treatment or no treatment) and fatal overdose (i.e., death without treatment). Second, because we relied on diagnostic codes to determine overdose episodes, we were unable to distinguish unintentional overdose from intentional overdose and could not confirm the specific drugs related to the overdose-related admissions. An additional limitation is the lack of information on the diagnostic accuracy for overdose and chronic conditions in the database. Third, the annual rate of overdose-related admissions in the present study may be underestimated because a suspected diagnosis of drug poisoning was not included in the analyses. Some patients without a definitive diagnosis of drug poisoning may receive supportive care with fluid resuscitation for the treatment of overdose.

## Conclusions

This nationwide epidemiological study provides the foundation for planning country- and region-specific overdose prevention programs. The findings of the present study emphasize the need for overdose prevention programs that focus on psychiatric patients aged 19–49 years with a prescription for benzodiazepines or barbiturates and on non-psychiatric patients aged ≥75 years with a prescription for benzodiazepines or digitalis.

## Conflicts of interest

During the past 3 years, YO has received research grants from the Japan Agency for Medical Research and Development; Ministry of Health, Labour and Welfare; Japan Society for the Promotion of Science; Institute for Health Economics and Policy; and Mental Health and Morita Therapy and served as a member of an advisory board for Janssen Pharmaceuticals, Inc. DN has received research grants from the Japan Society for the Promotion of Science, National Center of Neurology and Psychiatry, and Japan Agency for Medical Research and Development, and lecture fees from Otsuka Pharmaceutical Co. Ltd. HT receives research grants from the Japan Agency for Medical Research and Development and Ministry of Health, Labour and Welfare. The other authors declare no financial relationships with commercial interests.
