# Supplementary material for: Epidemiology of overdose episodes from the period prior to hospitalization for drug poisoning until discharge in Japan: An exploratory descriptive study using a nationwide claims database
Source: J Epidemiol. 2017 Feb 24;27(8):373–80. doi: 10.1016/j.je.2016.08.010 (PMC5549249; doi:10.1016/j.je.2016.08.010)
Supplement: Supplementary file 1 [file mmc1.pdf]

eTable 1. Complete list of psychotropic medications

Drug	Drug
Sedatives-hypnotics	Antipsychotics
alprazolam[a]	perospirone
amobarbital[b]	perphenazine
barbital[b]	pimozide
bromazepam[a]	pipamperone
bromovalerylurea[c]	prochlorperazine
brotizolam[a]	propericiazine
chloral[c]	quetiapine
chlordiazepoxide[a]	reserpine
chlorpromazine-promethazine-phenobarbital[b]	risperidone
clorazepate dipotassium[a]	spiperone
clotiazepam[a]	sulpiride
cloxazolam[a]	sultopride
diazepam[a]	tiapride
estazolam[a]	timiperone
eszopiclone[a]	trifluoperazine
ethyl loflazepate[a]	zotepine
etizolam[a]	Antidepressants
fludiazepam[a]	amitriptyline
flunitrazepam[a]	amoxapine
flurazepam[a]	clomipramine
flutazolam[a]	dosulepin
flutoprazepam[a]	duloxetine
haloxazolam[a]	escitalopram
hydroxyzine[c]	fluvoxamine
lorazepam[a]	imipramine
lormetazepam[a]	lofepramine
medazepam[a]	maprotiline
mexazolam[a]	mianserin
nimetazepam[a]	milnacipran
nitrazepam[a]	mirtazapine
oxazolam[a]	nortriptyline
pentobarbital calcium[b]	paroxetine
phenobarbital[b]	sertraline
phenobarbital sodium[b]	setiptiline
prazepam[a]	trazodone
quazepam[a]	trimipramine
ramelteon[c]	Mood stabilizers
rilmazafone[a]	carbamazepine
secobarbital sodium[b]	lamotrigine
tandospirone citrate[c]	lithium
tofisopam[a]	sodium valproate
triazolam[a]	Anticonvulsants

eTable 1. Complete list of psychotropic medications

Drug	Drug
triclofos[c]	acetazolamide
zolpidem[a]	acetylpheneturide
zopiclone[a]	bemegride
Antipsychotics	clobazam
aripiprazole	clonazepam
blonanserin	diazepam (DZP)
bromperidol	ethosuximide
carpipramine	ethotoin
chlorpromazine	fosphenytoin
clocapramine	gabapentin
clozapine	levetiracetam
fluphenazine	phenytoin
haloperidol	phenytoin-phenobarbital
haloperidol decanoate	primidone
levomepromazine	rufinamide
mosapramine	stiripentol
nemonapride	sultiame
olanzapine	topiramate
oxypertine	trimethadione
paliperidone	zonisamide
paliperidone palmitate	

[a] Benzodiazepines
[b] Barbiturates
[c] Other sedative-hypnotics.

eTable 2. Age- and sex-standardized rate ratios according to prefecture

Prefecture	n	Direct standardization[a]	Indirect standardization[b] (95% credibility interval)
Hokkaido	1,175	21.4	1.24 (1.17, 1.31)*
Aomori	168	12.1	0.77 (0.67, 0.88)*
Iwate	258	19.8	1.13 (1.01, 1.26)*
Miyagi	426	18.1	1.06 (0.96, 1.16)
Akita	192	18.2	1.04 (0.91, 1.18)
Yamagata	182	15.8	0.94 (0.82, 1.06)
Fukushima	347	17.5	1.02 (0.92, 1.13)
Ibaraki	622	21.4	1.24 (1.15, 1.34)*
Tochigi	366	18.8	1.09 (0.99, 1.20)
Gunma	404	20.5	1.19 (1.08, 1.30)*
Saitama	893	12.6	0.75 (0.70, 0.80)*
Chiba	1,123	18.5	1.09 (1.02, 1.15)*
Tokyo	2,487	18.2	1.08 (1.04, 1.12)*
Kanagawa	1,456	16.2	0.95 (0.90, 1.00)*
Niigata	343	14.4	0.87 (0.78, 0.96)*
Toyama	212	19.6	1.13 (0.99, 1.27)
Ishikawa	193	16.6	0.99 (0.86, 1.12)
Fukui	134	17.0	1.00 (0.86, 1.15)
Yamanashi	148	17.4	1.02 (0.88, 1.17)
Nagano	512	24.0	1.37 (1.26, 1.49)*
Gifu	277	13.5	0.82 (0.73, 0.91)*
Shizuoka	534	14.5	0.86 (0.79, 0.93)*
Aichi	905	12.4	0.74 (0.69, 0.79)*
Mie	243	13.4	0.81 (0.72, 0.90)*
Shiga	216	15.6	0.93 (0.82, 1.05)
Kyoto	381	14.5	0.86 (0.78, 0.95)*
Osaka	1,353	15.5	0.90 (0.86, 0.95)*
Hyogo	874	15.8	0.93 (0.87, 0.99)*
Nara	321	23.4	1.31 (1.18, 1.44)*
Wakayama	171	17.1	1.02 (0.88, 1.16)
Tottori	106	18.2	1.05 (0.89, 1.23)
Shimane	158	22.6	1.23 (1.07, 1.41)*
Okayama	317	16.3	0.97 (0.87, 1.07)
Hiroshima	571	20.2	1.18 (1.09, 1.27)*
Yamaguchi	222	15.5	0.92 (0.81, 1.04)
Tokushima	94	12.0	0.78 (0.65, 0.91)*
Kagawa	167	17.3	1.01 (0.88, 1.15)
Ehime	226	15.7	0.95 (0.84, 1.06)
Kochi	129	16.7	1.00 (0.85, 1.16)
Fukuoka	1,076	21.2	1.23 (1.16, 1.30)*
Saga	142	16.8	0.99 (0.86, 1.14)

Nagasaki	242	17.2	1.01 (0.89, 1.13)
Kumamoto	339	18.8	1.09 (0.98, 1.20)
Oita	214	18.2	1.05 (0.92, 1.18)
Miyazaki	157	14.3	0.86 (0.74, 0.98)*
Kagoshima	297	17.6	1.02 (0.92, 1.13)
Okinawa	290	21.8	1.22 (1.09, 1.35)*

[a] Direct standardization, age-sex standardized rate per 100,000 population in a prefecture.
[b] Indirect standardization, age- and sex-standardized rate ratio in a prefecture in comparison with that in Japan as a whole.
* indicates statistical significance.
